# Social networks as tools for the prevention and promotion of health among youth

**DOI:** 10.1186/s41155-020-00150-z

**Published:** 2020-07-15

**Authors:** José Antonio García del Castillo, Álvaro García del Castillo-López, Paulo César Dias, Fernando García-Castillo

**Affiliations:** 1 Universidad Miguel Hérnandez, San Joan D’Alacant, Spain; 2 Universidade Católica Portuguesa, Braga, Portugal; 3 Universidad de Alicante, Alicante, Spain

**Keywords:** Social networks, Prevention, Health promotion, Youth

## Abstract

The emergence of information and communication technologies (ICT) has generated a number of research questions, related to their use and potential risk, but also potentials for prevention or health promotion. Online social networks have become an important source of information for users as well as a tool for social relations. As traditional social networks, they can act as vehicles to improve the health of adolescents and youth, as well as play a key role in an educational context. The aim of this work is then to explore the theoretical relevance of ICT, particularly on online social networks, on disease prevention and health promotion of communicable diseases. Literature review points out the role of online social networks, particularly in the field of sexual health, body image, especially eating habits and overweight, as well as smoking and alcohol dependence. Data allow us to understand how online social network behavior and interaction is related to their burden and interventions developed in sexual health and addiction show positive results. More efforts in body image are needed in order to use these tools for prevention and promotion of health from early age.

## Introduction

The rapid evolution of new information and communication technology (ICT) over the past two decades has resulted in changes in lifestyles and a distinct social reality (García del Castillo, López-Sánchez, Tur-Viñes, García del Castillo-López, & Ramos, (2014a). This should come as no great surprise, given that there has been knowledge of this changing technology for some time. In fact, one of the first visionaries of these technologies and their influence on human behavior was Marshall McLuhan, who presented an incipient type of online social network in the 1960s: “… In an electric information environment, minority groups can no longer be contained – ignored. Too many people know too much about each other. Our new environment compels commitment and participation. We have become irrevocably involved with, and responsible for, each other” (McLuhan, (1967), p. 24).

From a more critical perspective, Carr (2013) suggested that technologies are being used by humans to gain control over our circumstances, that is, over nature, time and distance, and over others. Particularly, these technologies might allow us to complement or extend innate human capacities to increase physical capacity (e.g., from a sewing needle to a combat aircraft), to increase sensory capacity (e.g., from a microscope to a Geiger counter), to change nature to meet human needs (e.g., from a reserve to an oral contraceptive pill), or to expand or reinforce human mental capacity (e.g., from a typewriter to the Internet). However, based on a literature review, this author discusses the potential of technologies, particularly the internet, with respect to the brain, memory, and reading. In a provocative text, using several paradoxes, this author integrates data from the literature to challenge readers to evaluate their digital practices and reflect on the effect of these instantaneous information sources on our abilities.

In fact, as history has revealed, the search for new technologies is an ongoing attempt to improve our quality of life. The Internet was originally created for the military, but it was not developed or used for these purposes. Its creation coincides with the arguments of McLuhan, since it was ready for use by the late 1960s, but as Castells (2000) noted, it was not until the 1990s that the World Wide Web truly began to function. First, it served as a repository of documents and knowledge Carvalho, (2008); then, with the introduction of the so-called web 2.0 (O’Reilly, 2005), it was possible for every user to produce information and knowledge, edit works, and interact with each other Bebensee, Helms, & Spruit, (2011).

Social networks were one of these tools that were created and developed with web 2.0, but their impact on the global population has already been quite significant, and they have influenced all areas of life, from interpersonal relationships to the scientific realms. According to Boyd and Ellison (2008, 2008), social networks are online communities where it is possible to generate individual profiles and express any type of information to interact with other users, such that, in addition to communicating with real-life friends, it is also possible to meet others who share similar interests and hobbies.

According to Pintado and Sánchez (2017), social networks are based on two main axes that maintain them active: the creation of new content and social relationships. Many advantages of these networks include the ease of initiating real-time contact with millions of users across the world and the access to information and resources for people in need Bargh & McKenna, (2004; Lee & Cho, (2019); Valkenburg & Peter, (2007). The main disadvantages of social networks are their potential to become highly addictive (Andreassen, (2015); Brezing, Derevensky, & Potenza, (2010); García del Castillo et al., (2014a); Kuss & Griffiths, (2017); Young, (2010) or their potential to facilitate other mental health problems (Andreassen et al., (2016); Keles, McCrae, & Grealish, (2020); Pantic, (2014); Riehm et al., (2019). Studies have pointed out that adolescents and youth face particular risks Durkee et al., (2012); Kuss, van Rooij, Shorter, Griffiths, & van de Mheen, (2013); Riehm et al., (2019). Adolescents face challenges with respect to physical, psychological, and interpersonal development (Crosnoe & Johnson, (2011); Steinberg & Morris, (2001), such as a reconfiguration of their relationships with family and peers as well as pressure to assume vocational decisions Zimmer-Gembeck & Skinner, (2008). This is also a period that has been associated with the onset of risk behaviors (Defoe, Dubas, & Romer, (2010) that are maintained into adulthood and very often co-occur (Champion et al., (2019). Recent studies also highlight the links between social media use and risky behaviors during adolescence Vannucci, Simpson, Ggnon, & McCauley, (2020), but longitudinal data allow us to understand that adolescents who report frequently using social media face higher risks of mental health problems Riehm et al., (2019).

Given this context, some authors point out the need for a public health agenda for social media (Pagoto, Waring, & Xu, (2019) and the need to improve the use of eHealth in disease prevention and health promotion Champion et al., (2019). Traditionally, health prevention and promotion measures have sought to transmit information as a means for achieving their objectives. Therefore, the use of online social networks as instruments for the dissemination of preventive and promotional information for healthy lifestyles is theoretically quite feasible. Campaigns directed towards the prevention of numerous communicable diseases through information provided via traditional media have been effective since they have been supported by social advertising to promote protection against these diseases García del Castillo, López-Sánchez, Tur-Viñes, García del Castillo-López, & Ramos, (2014b).

The main objective of this work is to analyze the relevance of ICT, with a special emphasis on online social networks and the information process used to promote and prevent health problems among youth, together with traditional social networks. From the perspective of traditional epidemiology, it is possible to understand how technologies might significantly reduce many communicable diseases via disseminating information through traditional media, but these technologies fail to advance sufficiently with regard to noncommunicable diseases. Thus, a critical evaluation of the state of the art is presented herein to promote universal environmental mechanisms for health promotion and prevention in the social network context using a strategy adapted from the so-called “social vaccines” Baum, Narayan, Sanders, Patel, & Quizhpe, (2009); Dasgupta, (2011). We intend to clarify related concepts to explore the role of online social networks in the behavior of young people and to explore the role of online social networks in health prevention and promotion.

## Health promotion and prevention

Health promotion and prevention are two concepts that are strongly linked to health education. The conceptualization of health promotion according to the International Conference on Health Promotion held in Ottawa (1986, p.1) is as follows: “Health promotion is the process of enabling people to increase control over, and to improve, their health. To reach a state of complete physical mental and social wellbeing, an individual or group must be able to identify and to realize aspirations, to satisfy needs, and to change or cope with the environment”. Subsequently, it was redefined as follows WHO, (1998), p.10: “…comprehensive social and political process, it not only embraces actions directed at strengthening the skills and capabilities of individuals, but also action directed towards changing social, environmental and economic conditions so as to alleviate their impact on public and individual health. Health promotion is the process of enabling people to increase control over the determinants of health and thereby improve their health”. Disease prevention, according to the WHO (1998), is a complementary concept. Despite being called “an ancient concept imbedded in customs, culture and religion” (Scrimshaw, White, & Koplan, (2001), p.4), prevention is typically assumed to encompass all behaviors and practices relating to protecting health and preventing risks. In the words of Startfield, Hyde, Gérvas, and Heath (2008), the scope of health prevention has changed over time. It started as a set of strategies of activities to limit the progression of a disease Clark & McMahon, (1967), and it evolved with the work of Nightingale, Cureton, Kalmar, and Trudeau (1978) into primary prevention (i.e., prevention prior to the development of the disease) from secondary prevention (i.e., practices and strategies implemented in an earlier phase of the disease) and tertiary prevention (i.e., practices to lessen the impact of a disease or to try to reverse or delay disease progression).

The interaction of both concepts within a scholastic and academic context is in line with the concept of health education. According to Salvador and Suelves (2009), health education combines a set of opportunities for learning that implies the improvement of health-related information and the promotion of those skills that are required to improve health at individual levels and collective levels. According to the Instituto Proinapsa-UIS (2014), integral health education is a process involving the creation of new learning methods for self-care and collective care based on communication processes, among others.

If the advancement of epidemiology allows us to significantly reduce the incidence of many communicable diseases, then noncommunicable diseases will continue to require considerable attention (World Health Organization, (2018). Noncommunicable diseases, i.e., chronic diseases that are not spread or contagious, tend to result in a combination of generic, physiological, environmental, and behavioral factors, such as cardiovascular diseases or diabetes; communicable diseases (e.g., the flu, AIDS) are able to be spread from one person to another, whether that spread occurs via direct contact, wind, or water. According to Gershenson and Wisdom (2013), fighting infectious diseases may be quite simple since they are caused by a single cause: a single bacteria, parasite, or virus. Chronic and degenerative illnesses, such as cancer, diabetes, liver disease, and kidney disease, a priori, are not considered to be communicable; however, recently, research has suggested that unhealthy behavior might contribute to their burden Schwamm, (2018). These diseases are not communicable, but lifestyle factors may lead to a higher risk of developing the disease, and such factors have been shown to spread through one’s social networks of individuals. As an example, in a longitudinal analysis from 1971 to 2002, Christakis and Fowler (2007) found that individual obesity was correlated with social networks. They found a 37% to 57% increase in the likelihood of being obese if participants had a spouse, sibling, or friend that became obese in a given interval. These authors consider that the lifestyles of sick individuals can be “transmitted” to those in their nearby environment (family members, friends, colleagues, etc.). According to Christakis and Fowler (2013), the social contagion theory suggests that online social influence might be transmitted by an individual up to three degrees away. With an example presented by the authors, it might be possible to verify a correlation between an individual’s body mass index and that of his or her friends (first degree), his or her friends’ friends (second degree), and their friends’ friends (third degree). Online social networks that provide social contact with multiple contacts significantly increase the speed of contagious behavioral thoughts about the population Zhang & Centola, (2019). If, as Dunbar (2010) suggested, online social network users have an average of 150 potential friends and if, as stated, this influence spans three degrees, this means some three million individuals could possibly influence everyone. It is important, then, to understand the relationships between online social networks and youth health as well as how these tools can be used for heath prevention and promotion.

## The influence of social networks on the health of youth

The massive use of social networks and instant messaging by young people results in two possibilities. On the one hand, social networks increase the quantity of opportunities for interacting with individuals from across the globe, the ability to access social support or to help to create one’s own identity; on the other hand, social networks increase the number of potential dangers, such as those resulting from cyberbullying or real encounters with strangers, among many others.

Clearly, education and training in the area of social networks continue to require improvement, but there are indications of their potential in terms of health promotion and prevention. According to Arab and Diaz (2015), the positive situations resulting from the use of social networks by youth may be based on the following aspects:

- The maintenance of in-person relationships
- The exploration of one’s own identity through relationships with peers
- Finding support for problems
- Developing new skills

The following negative situations should be considered:

- The development of relationships with strangers
- The risk of addiction
- The increased probability of being a victim of cyberbullying (sexting, cyberbullying, grooming, etc.)

According to Smith and Christakis (2008), three categories in the areas of health have a greater influence and repercussion on young users of social networks: sexual health, body image (e.g., eating habits and excess weight), and addictions (mainly smoking and alcohol use). Thus, it is important to explore relationships between these areas and online social network use and to explore online health promotion and prevention efforts toward these issues.

### Sexual health

During adolescence, the basic structure of human sexuality is developed, and without a doubt, this is the most important period for the development of good sexual health. In human evolutionary development, at the age of 11 or 12, a cycle of body transformations and the discovery of one’s own body occur. Later, between the ages of 13 and 15, experimentation begins, and between 16 and 20, a more meditated sexuality develops, including the development of emotional ties Estefenon & Eisenstein, (2015).

Given the role of social media in youth, some studies have pointed out some risks with self-esteem and suggested the promotion of new social norms and beliefs regarding risky sexual behaviors Cookingham & Ryan, (2015); Eckstrand et al., (2017). Particularly, exposure to sexual content, especially in the media (including social media), appears to be a predictor of adolescents’ sexual behavior Vandenbosch, van Oosten, & Peter, (2015). As the authors mention, initial relations may begin over social networks with known or unknown individuals, entering into what may be called an “online lifestyle”, resulting in the loss of intimacy and privacy Estefenon & Eisenstein, (2015).

This new online lifestyle plays a major role in body image creation. According to Peris, Maganto and Kortabarria (2013), adolescents of both sexes having greater social and erotic self-esteem tend to upload more esthetic and erotic photographs online. However, males are found to have greater self-esteem compared to females and more strategies for sexual and emotional advances that are more positive in the face of sexuality; on the other hand, females are more likely to upload a greater number of esthetic and erotic photographs on social networks.

Adolescents of both genders tend to be reckless, violate their own intimacy and privacy, and upload previously edited esthetic and erotic photographs along with bold textual content Maganto & Peris, (2013). Generally, the use of social networks for the development of sexuality and the creation of body image, among other issues, is essential for this new adolescent generation (Escobar & Román, (2011); Menjívar, (2010); Mitchell, Finkelhor, Jones & Wolak, (2012).

The construction of sexuality in the digital era brings with it certain issues to be considered (Estefenon & Eisenstein, (2009), of which the following are the most representative:

- In terms of normal development:

- Bodily changes during puberty
- The need for sexual experimentation
- The creation of the self-image and self-esteem
- The need for affection
- The creation of one’s own identity

- In terms of intervention on social networks:

- Sexual misinformation
- Depersonification of relations
- Sexting
- Dissemination of privacy
- Socialization with strangers
- Double personality: online and real

Clearly, Internet and social network mediation has led to significant variation in the evolution of sexual development in youth. However, some seminal research has also highlighted the role of social media in the promotion of sexual health. In a review study, Gabarron and Wynn (2016) reviewed 51 observational, nonrandomized and randomized intervention studies on the promotion of sexual health. Despite some criticism of theoretical or methodological issues, evidence suggests that there is a positive effect of social media on the promotion of sexual health. A more recent study about interventions found the same positive results for sexual health outcomes in the short (< 6 months) and long term (> 6 months) (Hunter et al., (2019). Additionally, studies in which health practitioners use social media messages to promote adequate sexual health information for youth have shown positive results (e.g., Stevens, Gilliard-Matthews, Dunaev, Todhunter-Reid, Brawner, & Stewart, (2017). Assuming that adolescents tend to relate with peers with the same experience (McCann, Broccatelli, Moore, & Mitchell, (2018), the dissemination of accurate and appropriate information to adolescents seems crucial.

### Body image: eating habits and excess weight

For youth and adolescents, body image is a primary concern (Vilhjalmsson, Kristjansdottir, & Ward, (2011); Voelker, Reel, & Greenleaf, (2015). These individuals tend to believe that their body image leads to the success or failure of their interpersonal relationships, which becomes their main objective. Body image includes various dimensions (Ahrber, Trojca, Nasrawi & Vocks, (2011), as follows:

- Perceptive dimension: how one sees oneself
- Cognitive–affective dimension: construct of thoughts and emotions directly related to one’s own body
- Behavioral dimension: actions caused by what one thinks and feels, translating to verifications of weight, measurements, etc.

In an interesting study by Escandón, et al. (2019), the authors observed gender differences with regard to body image in young adults. It was seen that females had lower self-esteem when they perceived themselves as being heavier/more overweight. The authors referred to this as discrepancies between how they perceive themselves and how they would like to be perceived (the real me vs the ideal me). Generally, women tend to link body image to self-esteem, aspects of their own identity and sensations of social isolation (feeling different from others in their social environment). Men, on the other hand, do not typically associate body image with self-esteem.

Regarding eating habits as a high-risk factor in youth, at-risk eating behavior and eating disorders are of special relevance, with both of these currently existing in a small proportion of youth and adolescents. At-risk eating behavior may lead to eating disorders, which have the same characteristics but with a greater frequency and intensity.

At-risk eating behaviors share a series of associated behaviors (Unikel, Bojórquez, & Carreño, (2004):

- Sensation of loss of control with eating
- Recurrent and irrational concern over gaining weight
- Behavior associated with diets and other food restrictions

Social networks play a very significant role in terms of body image and eating behavior. A significant correlation between hours spent on social networks and at-risk eating behaviors has been found (Caldera, Martín del Campo, Caldera, Reynoso & Zamora, (2019); Santarossa & Woodruff, (2017). Additionally, experimental and review studies have highlighted a correlation between internet use, especially when related to image and appearance, and the internalization of body image (Hogue & Mills, (2019); Mingoia, Hutchinson, Wilson, & Gleaves, (2017) as well as body image and eating concerns (Rodgers & Melioli, (2016). A mixed methods systematic review (Rounsefell et al., (2020) identified correlations between social media engagement or exposure to content related to body image and body dissatisfaction, dieting, overeating, and choosing healthy foods, with qualitative data emphasizing the role of comparison, modifying one’s appearance to portray an ideal image, and external validation. Despite these data and the role of body image in youth behaviors, no related interventions were found in the literature.

### Addictions: smoking, and alcohol

**Fig. 1:**
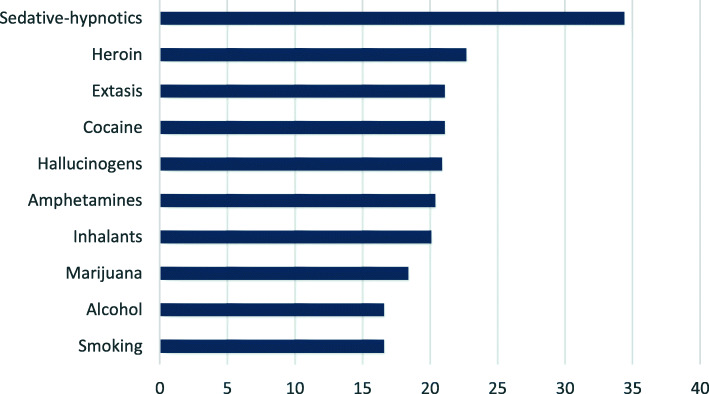
Age of onset for consumption in the population aged 15-64 in Spain. Source: PNsD, (2017)

Substance addiction is one of the main health problems of the general population, especially among youth. Alcohol use and smoking (both of which are legal activities) are the most common addictions, and the age of onset is approximately 16 years old. Variability in the age of onset for the consumption of other substances ranges from 18 years of age for marijuana to approximately 34 for sedative-hypnotic drugs (PNsD, 2017) (Fig. 1). A similar trend is also found in Europe and across the world, indicating the need for attention and prevention efforts.

The risk factors for the onset of consumption are very closely related to peer pressure and social factors in general, with social networks playing an especially important role (Navarro-Oliva, Anda, Gámez, Candía & Guzmán, (2016). Regarding the influence of social networks on the consumption of these substances, two distinct approaches should be considered (Galván, Serna & Hernández, (2008):

- Consumption is directly related to impact: peer pressure, consumption by family members and friends, and consumption risk factors
- Consumption is related to the characteristics of the networks used to keep track of the level of influence at the onset of consumption and during the subsequent maintenance of consumption

Some authors (Cruz, Montero, Salas & Ortiz, (2016); Montero, Cruz, Tiburcio & García, (2018) consider the content related to alcohol, smoking, and other drugs disseminated by social networks as a primary source of information. Three sources are mentioned:

1. Publications that network contacts upload: photographs and/or videos of parties with alcohol, smoking, and other drugs
2. Web pages and independent users who upload content referring to alcohol, smoking, and other drugs where decriminalization and benefits are considered, omitting any problems
3. Advertisements for alcohol and smoking included in distinct social networks

Finally, a significant relationship has been observed between exposure to alcohol, smoking, and other drug content over social networks and consumption in adolescents (Lerma, Yáñez, Sosa, Medina, Villegas & Vargas, (2017); Navarro-Oliva, Anda, Gámez, Candía & Guzmán, (2016). However, some authors point out the potential role of social networks in the prevention of alcohol use (Moreno, D’Angelo, & Whitehill, (2016), while others present different kinds of interventions, mostly focused on adults (Kennedy, Hunter, Osilla, Maksabedian, Golinelli, & Tucker, (2016), but the results tend to be nonsignificant Hunter et al., (2019). Some recommendations have been published Costello & Ramo, (2017): (i) incorporate social media use in screenings and assessment procedures; (ii) encourage teens to discuss substance use beliefs and thoughts that they are exposed to via social media information or friends; (iii) implement familiar social media policies at home that respect autonomy but also allow some parental monitoring; and (iv) evaluate parents’ knowledge about social media activities. However, additional intervention programs and evaluation studies are needed.

## Conclusions

Many authors have proposed the use of social networks as instruments for health prevention and promotion. Considering the potential of social networks in health behavior Fowler & Christakis, (2011), the detrimental role of social networks in noncommunicable diseases and risky behaviors should be researched. In this paper, by exploring three of the main risk factors for noncommunicable diseases in adolescence and youth, the authors highlighted the role of social networks in the onset and development of these problems and the role of social networks in health prevention and promotion. The literature shows a tendency for positive outcomes in sexual health prevention and promotion, while much research and intervention is needed to promote adequate body image and to prevent addiction. Regarding good practices to foster health prevention and promotion actions, it is necessary to test the efficacy of the following dimensions in offline social networks Fernández, (2005):

- Size: Increasing the number of individuals making up a social network where the main subject maintains his/her contacts is positively related to health and wellbeing
- Density: Increasing the density (interconnections) of a social network leads to a greater probability of emotional support for an individual, thereby affecting his/her health and quality of life
- Reciprocity: Increasing the reciprocity of network resources promotes health states. Reciprocity indicates that the individual does not always initiate contact
- Homogeneity: Increasing the similarity and congruence between members of the social network improves health expectations

Therefore, it is important to promote new research and practices to allow adolescents not only to have access to information but also to share their doubts, dilemmas, and questions with specially trained professionals without the intention to substitute the role of educators or psychologists but as a counseling strategy for a generalized target population. Existing studies point out some positive results of such approaches.

It is necessary to develop behavioral changes to address excess weight and obesity among youth. The content of alcohol, smoking and other drug consumption in social networks and on the Internet is positively associated with consumption in adolescents. Social networks must be included as a fundamental variable for interventions in health prevention regarding these substances, and online interventions have yet to produce significant results. Thus, it is important to develop new theoretical and developmentally driven tools to prevent disease and promote health in youth.

The use of online social networks is almost ubiquitous in the youth population. As in the educational field, these tools initially generated significant concerns and fears, but increasingly, they have been introduced in teaching practices. Additionally, research is beginning to examine the use of social networks in health prevention and promotion. Further studies are necessary to promote universal information and skills as “social vaccines” in youth. As vaccines are an important instrument for the prevention of illness, health promotion and prevention are determinants to encourage population mobilization and advocacy toward health (Baum, Narayan, Sanders, Patel, & Quizhpe, (2009); Dasgupta, (2011). This is an important movement to promote children’s and youth’s rights to integral education, to promote the necessary skills to interact socially and to promote health and wellbeing at early ages. For the development of these interventions, involving young people as coresearchers in the development of specific interventions might be useful for designing a specific social network for health promotion.

## Data Availability

Given the nature of the paper (review paper), there are no data and materials.
